# Headspace Single-Drop Microextraction Gas Chromatography Mass Spectrometry for the Analysis of Volatile Compounds from Herba Asari

**DOI:** 10.1155/2013/380705

**Published:** 2013-03-28

**Authors:** Guan-Jie Wang, Li Tian, Yu-Ming Fan, Mei-Ling Qi

**Affiliations:** ^1^National Institutes for Food and Drug Control, No. 2, Tiantan Xili, Beijing 100050, China; ^2^Beijing Institute of Technology, No. 5, Zhongguancun South Street, Haidian District, Beijing 100081, China

## Abstract

A rapid headspace single-drop microextraction gas chromatography mass spectrometry (SDME-GC-MS) for the analysis of the volatile compounds in Herba Asari was developed in this study. The extraction solvent, extraction temperature and time, sample amount, and particle size were optimized. A mixed solvent of n-tridecane and butyl acetate (1 : 1) was finally used for the extraction with sample amount of 0.750 g and 100-mesh particle size at 70°C for 15 min. Under the determined conditions, the pound samples of Herba Asari were directly applied for the analysis. The result showed that SDME-GC–MS method was a simple, effective, and inexpensive way to measure the volatile compounds in Herba Asari and could be used for the analysis of volatile compounds in Chinese medicine.

## 1. Introduction

Single-drop micro-extraction (SDME), a new sample preparation technique introduced by Jeannot and Cantwell [1], has attracted increasing attentions. In this straightforward technique, a microdrop of solvent is suspended at the tip of a conventional microsyringe and then exposed in the sample headspace. Since only the volatile or semivolatile compounds can be volatilized into headspace and extracted to a single drop, interferences from the complex matrix will be decreased greatly. Other advantages of this method are the small amount of organic solvent required and the simple experimental and sampling equipment, while extraction, concentration, and sample introduction are integrated into a single step [2]. Several papers have been published using this sample preparation approach for the determination of environmental specimen [3–8], food [9, 10], biological products [11–14], engine oil samples [15], and pesticide residue [10, 16]. In SDME process, it is very important to use a suitable extraction solvent to achieve a good selectivity for the analytes, especially for complicated samples. In the above-mentioned papers, only one extraction solvent for several substances was employed [3–16], but there is little reported for complex extraction solvent. In our previous investigations, a single extraction solvent was successfully used 

to analyze traditional Chinese medicine (TCM) [17, 18]. However, these methods based on a single extraction still had the following drawbacks that amount and categories of extracted compounds almost depended on the polarity of the single extraction solvent.

To overcome the shortcomings of single solvent extraction in the present study, mixed solvent extraction based on SDME was developed for analysis of volatile components in TCM, Herba Asari. Herba Asari was used to dispel wind heat, headache, toothache, snuffling, and rheumatism [19]. The method precision and the parameters of SDME were studied. 

## 2. Experimental

### 2.1. Reagents and Materials

The extraction solvents (benzyl alcohol, n-tridecane, n-tetradecane, 1,4-butanediol, butyl acetate, n-dodecane, methylbenzene, 1-octanol, decane, decanol, isobutyl alcohol, and n-tridecane) were purchased from our Chem Company (of GC grade or minimum purity of 99%) and used without any further purification. One-microliter SGE microsyringe and water bath were purchased for the SDME procedure. A manual SPME holder and 100 *μ*m PDMS fibers from Supelco (Bellefonte, PA, USA) were used for the SPME procedure. Fibers were conditioned prior to use according to the supplier's instructions.

The dry root samples of Herba Asari were purchased from An'guo traditional Chinese medicine market in Hebei province of China and were authenticated by the Institute of Medicinal Plants, Academy of Medical Science of China. The samples were dried in air, cut, milled, and then sieved via 40, 60, 80, 100, and 120 mesh, respectively, to obtain different particle size samples. Finally, the pound samples were stored in tightly sealed weighing bottles until analysis.

### 2.2. GC-MS Conditions

Chromatographic separation was performed on an Agilent 6890 GC (USA) with a PEG-20W Innowax (Agilent) capillary column (30 m × 0.25 mm × 0.25 *μ*m). The oven temperature program was 7.0 min at 50°C 20°C/min to 130°C (keeping 130°C for 1 min), 1°C/min to 150°C, and 8°C/min to 190°C (keeping 190°C for 5 min). The injector and detector temperatures were 230°C and 260°C, respectively. Nitrogen of high purity was used as the carrier gas at a flow rate of 1.0 mL/min. The split ratio was 1 : 100. The mass spectrometer was fitted with an EI source operated at 70 eV, and mass spectra were recorded in the range of *m/z* 50 to 450 amu in the full-scan acquisition mode. The interface temperature and the ion source temperature were fixed at 240°C and 230°C, respectively. Volatile compounds were identified by comparing the obtained mass spectra of the analytes with those of authentic standards from the NIST and Hist 98 libraries with a resemblance percentage above 85%.

### 2.3. SDME Procedure

A 15 mL vial (Supclo, USA) with PTFE septum containing the powdered sample was placed at a fixed position in a water bath. Then a 1 *μ*L GC micro-syringe was pierced into the headspace of the vial, which was clamped at a fixed position for improving precision of the method. The microsyringe was washed at least 20 times by extraction solvent between runs. After a preset extraction time, the extraction solvent was retracted into the needle and swiftly injected onto GC-MS for the analysis.

SDME parameters including the type of the extracting solvent, extraction temperature and time, headspace volume (sample amount), solvent volume, and particle size of the pound sample were investigated.

## 3. Results and Discussion

### 3.1. Selection of SDME Conditions

**Figure 1:**
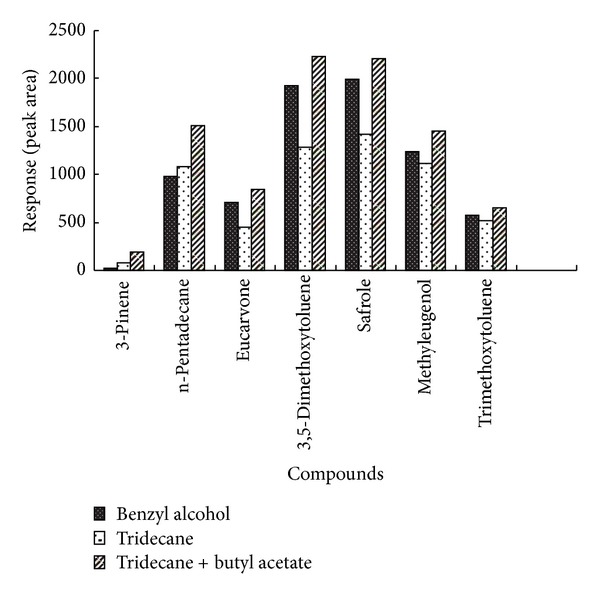
Comparison among different extraction solvents on the relative peak area of the seven main compounds from Herba Asari. Powdered sample, 0.500 g; extraction solvent volume, 0.6 *μ*L; particle size, 100 mesh; extraction time, 20 min, extraction temperature, 70°C; the percent between two mixed solvent, 1 : 1 (volume).

Selecting a proper extraction solvent is especially crucial for the analysis of volatile compounds of TCMs because of the great differences of the compounds in polarity and volatility. Mass transfer of the analytes from the pound sample to the microdrop continues until thermodynamic equilibrium is attained or the extraction is stopped according to the considerations of SDME. The principle “like dissolves like” can become the basis of the solvent selection. Different solvents were tested to find a suitable one that meets such requirements as high extraction efficiency in terms of the compounds and yields especially those of low volatility, less toxicity, and satisfactory chromatographic resolution for the analytes. Low volatility is helpful to keep the solvent micro-drop at the tip of the micro-syringe needle sustainable over the extraction time period. If possible, the front/end solvent peak is preferred to avoid the solvent problem with GC-MS. The decane, n-tridecane, n-tetradecane, butyl acetate, methylbenzene, 1-octanol benzyl alcohol, decanol, and isobutyl alcohol were selected and benzyl alcohol offered a better extraction. Then a mixture of two solvents was selected and after a detailed comparison of the peak areas of the seven main compounds (shown in Figure 1), n-tridecane mixed with butyl acetate was found to be the optimal combination and was finally adopted as the extraction solvent.

### 3.2. Percent of Mixed Extraction Solvent

**Figure 2:**
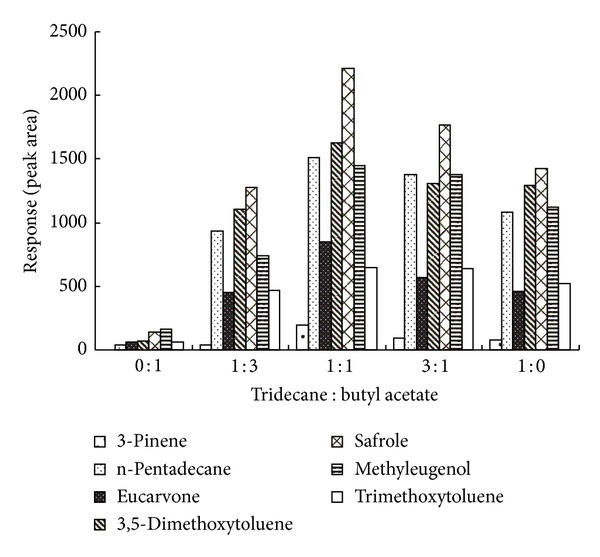
Comparison among different scales of n-tridecane in the extracting solvent on the relative peak area of the seven main compounds from Herba Asari. Powdered sample, 0.500 g; extraction solvent volume, 0.6 *μ*L; particle size, 100 mesh; extraction time, 20 min, extraction temperature, 70°C; the percent between two mixed solvent, 1 : 1 (volume).

Selecting a proper percent of mixed extraction solvent is also important for the analysis of volatile compounds. The volume scale of the mixed solvent of n-tridecane and butyl acetate was investigated in the ranges of 1 : 0, 3 : 1, 1 : 1, 1 : 3, and 0 : 1, while keeping the other parameters under the following conditions: powdered sample, 0.500 g; extraction solvent volume, 0.6 *μ*L; particle size, 100 mesh; extraction time, 20 min; extraction temperature, 70°C. The results showed that the peak area ratios of analytes of interest to benzyl alcohol (internal standard 0.2%) rapidly increased with the elevated percent of n-tridecane from 0 to 50 but decreased slowly after 50% n-tridecane. As a result, 50% was chosen as the extraction percent for the analysis (show in Figure 2).

The solvent volume was investigated by setting the volume of the solvent at the volume of the solvent in the range of 0.30 to 0.90 *μ*L. It can be known that the amount of the extracted analytes in the solvent drop increased with the solvent volume. However, the results also showed that when the volume exceeded 0.60 *μ*L, the chromatographic peak of the solvent broadened and even overlapped the peaks of analytes of interest. In light of this, the solvent volume of 0.60 *μ*L was finally used in the present study.

### 3.3. Extraction Time

The extraction time was determined by varying the exposure time of the microdrop in the headspace of a sample from 5 to 25 min while keeping the other parameters under the same conditions as Section 3.2. The peak areas of the seven main chosen compounds to the peak areas of benzyl alcohol were different from 5 to 25 min, but their sum peak areas were the largest at 15 min. So the extraction time of 15 min was chosen for the present work.

### 3.4. Extraction Temperature

The extraction efficiency of SDME procedure was temperature dependent. The effect of sample temperature on the extraction efficiency was investigated in the range of 50–90°C while keeping the other parameters under the same conditions as Section 3.3. The results showed that the peak areas of the seven main compounds in Herba Asari and their sum peak areas to benzyl alcohol (internal standard 0.2%) increased significantly with the temperature from 50°C to 70°C but deceased dramatically after 70°C. As a result, 70°C was chosen as the extraction temperature for the analysis.

### 3.5. Particle Size of Sample

For a solid sample, particle size plays an important part in the extraction. Particle size of the sample was tested from 40 to 120 mesh at 70°C for 15 min with powdered sample 0.500 g. The results showed that the amount of volatile compounds increased with the decrease of particle size from 40 to 100 mesh but deceased after 100 mesh. As a result, 100 mesh was chosen as the optimal extraction particle size of the sample. 

### 3.6. Sample Amount

Sample amount was determined by varying the amount of the powdered sample in a 15 mL vial. Increasing sample amount led to an increase of the analytes concentrations in the headspace which chanced the extracted amount of the analytes in the drop and a decrease of headspace volume. The extracted amount of the analytes increased continuously with the sample amount from 0.25 to 0.75 g and then decreased at 1.25 g. This observation could be explained by the fact that the powdered sample matrix could not be stirred during the process; consequently, with the increase of the sample amount, both the convection in the matrix and the mass transfer became slow. So 0.75 g was chosen as the optimal sample amount.

### 3.7. Repeatability

Under the determined conditions, the repeatability of the SDME method was determined by performing six replicate experiments. Relative standard deviations (RSD) of the seven main peak areas of the analytes of interest to the internal standard were 2.3% for 3-Pinene, 8.7% for eucarvone, 7.5% for 3,5-dimethoxytoluene, 9.2% for n-pentadecane, 9.8% for safrole, and 9.0% for methyleugenol. The RSD values for the seven compounds were all below 9.8%, indicating a satisfactory repeatability of the SDME method compared with the above-mentioned papers 10.8% [17]. 

### 3.8. SDME-GC-MS Results

**Figure 3:**
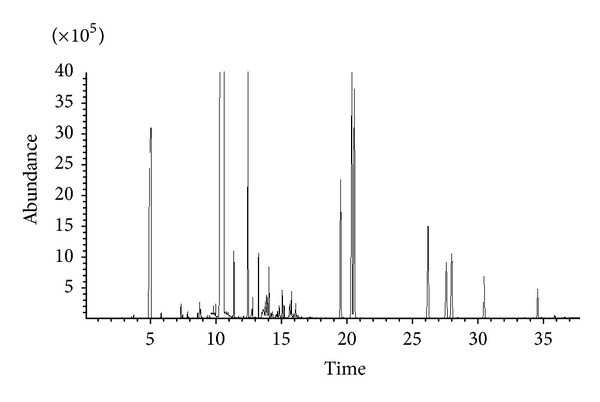
Typical chromatograms of volatile components from Herba Asari by SDME-GC-MS.

The typical total ion chromatograms of SDME-GC-MS methods are shown in Figure 3 and the corresponding volatile compounds identified are listed in Table 1. The retention index of every compound was calculated under the same temperature process. The number of the volatile compounds identified was 61 for SDME-GC-MS. In general, SDME-GC-MS can be used as a good method for the analysis of volatile compounds in TCMs. SDME-GC-MS has a much lower cost (only using negligible volume of a solvent) and wider availability of extraction solvents.

## 4. Conclusions

SDME-GC-MS has a lower cost, more choices of extraction solvents, requires a smaller amount of sample, and directly utilizes the ground powder of traditional Chinese medicines for the analysis. SDME-GC-MS method is a simple, cheap, and effective method for the analysis of volatile compounds in TCM.

## Figures and Tables

**Table 1:** Components of Herba Asari obtained by SDME-GC-MS.

No	Retention time (min)	Compounds	RI	SDME- (mixed-) GC-MS RA%
1	3.60	Acetic acid, 2-methylpropyl ester	1047	0.07
2	3.76	1R-.alpha.-Pinene	1052	0.21
3	5.85	beta.-Pinene	1099	0.34
4	7.40	3-Carene	1158	0.83
5	7.88	.alpha.-Phellandrene	1172	0.28
6	7.94	beta.-Phellandrene	1174	0.01
7	8.65	D-Limonene	1192	0.21
8	8.88	Eucalyptol	1193	0.55
9	9.36	1,3,6-Octatriene, 3,7-dimethyl-, (E)-	1230	0.08
10	9.50	1,4-Cyclohexadiene, 1-methyl-4-(1-methylethyl)-	1240	0.10
11	9.61	1,3,6-Octatriene, 3,7-dimethyl-, (Z)-	1248	0.08
12	9.67	Nonane, 3-methyl-	1253	0.32
13	9.73	Tridecane, 7-methyl-	1257	0.51
14	9.85	Tridecane, 7-methyl-	1265	0.86
15	9.96	Benzene, 1-methyl-2-(1-methylethyl)-	1272	0.31
16	10.03	Cyclohexene, 1-methyl-4-(1-methylethylidene)-	1277	0.45
17	10.86	Cyclohexene, 1-methyl-4-(1-methylethylidene)-	1598	0.21
18	11.40	Tetradecane	1397	2.23
19	11.74	Benzene, 1-methyl-4-(1-methylethenmethylethyl)-	1428	0.08
20	12.16	1,3-Cyclohexadiene, 1-methyl-4-(1-methylethyl)-	1467	0.08
21	12.49	Pentadecane	1496	12.80
22	12.74	1-Hexadecanol	1516	0.20
23	12.77	Bicyclo[2.2.1]heptan-2-one, 1,7,7-trimethyl-, (1R)-	1518	0.34
24	12.82	Cyclopentadecane	1522	0.65
25	13.29	Eucarvone	1556	2.33
26	13.55	Aristolene	1574	0.43
27	13.63	1,6,10-Dodecatriene, 7,11-dimethyl-3-methylene-, (Z)-	1580	0.75
28	13.68	4,7-Methanoazulene,1,2,3,4,5,6,7,8-octahydro-1,4,9,9-tetramethyl-,[1S-(1.alpha.,4.alpha.,7.alpha.)]-	1584	0.31
29	13.80	Azulene,1,2,3,4,5,6,7,8-octahydro-1,4-dimethyl-7-(1-methylethenyl)-,[1S-(1.alpha.,4.alpha.,7.alpha.)]-	1592	1.07
30	13.90	1H-Cyclopropa[a]naphthalene,1a,2,3,5,6,7,7a,7b-octahydro-1,1,7,7a-tetramethyl-,[1aR-(1a.alpha.,7.alpha.,7a.alpha.,7b.alpha.)]-	1599	1.26
31	13.94	3-Cyclohexen-1-ol,4-methyl-1-(1-methylethyl)-, (R)-	1601	0.66
32	14.06	2H-2,4a-Methanonaphthalene,1,3,4,5,6,7-hexahydro-1,1,5,5-tetramethyl-, (2S)-	1608	0.32
33	14.20	1H-Cyclopenta[1,3]cyclopropa[1,2]benzene,octahydro-7-methyl-3-methylene-4-(1-methylethyl)-,[3aS-(3a.alpha.,3b.beta.,4.beta.,7.alpha.,7 aS*)]-	1616	0.27
34	14.29	Benzene, 1,3,5-tris(1-methylethyl)	1620	0.27
35	14.37	Thujopsene	1625	0.39
36	14.60	Benzoic acid, 2,4-bis[(trimethylsilyl)oxy]-, trimethylsilyl ester	1637	0.07
37	14.65	1H-3a,7-Methanoazulene,2,3,6,7,8,8a-hexahydro-1,4,9,9-tetramethyl-, (1.alpha.,3a.alpha.,7.alpha.,8a.beta.)-	1640	0.23
38	14.74	Androsta-1,4-dien-3-one,17-hydroxy-17-methyl-, (17.alpha.)-	1644	0.41
39	14.86	Cyclohexane, 1,4-bis(methylene)-	1651	0.83
40	14.98	1,6,10-Dodecatriene,7,11-dimethyl-3-methylene-, (Z)-	1657	0.05
41	15.08	Estragole	1662	1.40
42	15.22	Naphthalene,1,2,3,5,6,8a-hexahydro-4,7-dimethyl-1-(1-methylethyl)-, (1S-cis)-	1669	0.65
43	15.60	Heptadecane	1688	0.15
44	15.67	1H-3a,7-Methanoazulene,octahydro-3,8,8-trimethyl-6-methylene-,[3R-(3.alpha.,3a.beta.,7.beta.,8a.alpha.)]-	1692	1.11
45	15.69	3-Cyclohexene-1-methanol,.alpha.,.alpha.4-trimethyl-	1693	0.21
46	15.80	Borneol	1698	1.41
47	15.98	Azulene,1,2,3,3a,4,5,6,7-octahydro-1,4-dimethyl-7-(1-methylethenyl)-,[1R-(1.alpha.,3a.beta.,4.alpha.,7.beta.)]-	1706	0.31
48	16.11	Azulene,1,2,3,5,6,7,8,8a-octahydro-1,4-dimethyl-7-(1-methylethenyl)-,[1S-(1.alpha.,7.alpha.,8a.beta)]-.	1711	0.84
49	16.30	cis-.alpha.-Bisabolene	1719	0.17
50	16.55	1H-Cyclopropa[a]naphthalene,1a,2,3,5,6,7,7a,7b-octahydro-1,1,7,7a-tetramethyl-,[1aR-(1a.alpha.,7.alpha.,7a.alpha.,7b.alpha.)]-	1729	0.18
51	19.54	3,5-Dimethoxytoluene	1845	10.50
52	20.41	Safrole	1878	20.39
53	26.21	Benzene, 1,2-dimethoxy-4-(2-propenyl)-	2070	9.94
54	27.59	Methyleugenol	2110	6.29
55	27.99	Benzene, 1,2,3-trimethoxy-5-methyl	2121	7.33
56	30.48	1,3-Benzodioxole,4-methoxy-6-(2-propenyl)-	2188	5.11
57	33.58	Patchouli alcohol	2264	0.10
58	34.56	1,3-Benzodioxole,4-methoxy-6-(2-propenyl)-	2286	2.31
59	35.83	Benzene, 1,2,3-trimethoxy-5-(2-propenyl)-	2313	0.05
60	35.89	3-(4-N,N-Dimethylaminophenyl)propenoic acid, 2-(diethoxyphosphinyl)-, ethyl ester	2314	0.07

SUM				60
